# Regional differences in clinical care among patients with type 1 diabetes in Brazil: Brazilian Type 1 Diabetes Study Group

**DOI:** 10.1186/1758-5996-4-44

**Published:** 2012-10-29

**Authors:** Marília B Gomes, Roberta A Cobas, Alessandra S Matheus, Lucianne R Tannus, Carlos Antonio Negrato, Melanie Rodacki, Neuza Braga, Marilena M Cordeiro, Jorge L Luescher, Renata S Berardo, Marcia Nery, Maria do Carmo Arruda-Marques, Luiz E Calliari, Renata M Noronha, Thais D Manna, Lenita Zajdenverg, Roberta Salvodelli, Fernanda G Penha, Milton C Foss, Maria C Foss-Freitas, Antonio C Pires, Fernando C Robles, MariadeFátimaS Guedes, Sergio A Dib, Patricia Dualib, Saulo C Silva, Janice Sepulvida, Henriqueta G Almeida, Emerson Sampaio, Rosangela Rea, Ana Cristina R Faria, Balduino Tschiedel, Suzana Lavigne, Gustavo A Cardozo, Mirela J Azevedo, Luis Henrique Canani, Alessandra T Zucatti, Marisa Helena C Coral, Daniela Aline Pereira, Luiz Antonio Araujo, Monica Tolentino, Hermelinda C Pedrosa, Flaviane A Prado, Nelson Rassi, Leticia B Araujo, Reine Marie C Fonseca, Alexis D Guedes, Odelissa S Matos, Manuel Faria, Rossana Azulay, Adriana C Forti, Cristina Façanha, Ana Paula Montenegro, Renan Montenegro, Naira H Melo, Karla F Rezende, Alberto Ramos, João Sooares Felicio, Flavia M Santos, Deborah L Jezini, Marilena M Cordeiro

**Affiliations:** 1 Unit of Diabetes, Universidade Estadual do Rio de Janeiro, Avenida 28 de Setembro, 77, 3o andar, CEP 20.551-030, Rio de Janeiro, Brazil

**Keywords:** Type 1 diabetes, Glycemic control, Cardiovascular risk factors, Chronic complications, Economic status

## Abstract

**Background:**

To determine the characteristics of clinical care offered to type 1 diabetic patients across the four distinct regions of Brazil, with geographic and contrasting socioeconomic differences. Glycemic control, prevalence of cardiovascular risk factors, screening for chronic complications and the frequency that the recommended treatment goals were met using the American Diabetes Association guidelines were evaluated**.**

**Methods:**

This was a cross-sectional, multicenter study conducted from December 2008 to December 2010 in 28 secondary and tertiary care public clinics in 20 Brazilian cities in north/northeast, mid-west, southeast and south regions. The data were obtained from 3,591 patients (56.0% females and 57.1% Caucasians) aged 21.2 ± 11.7 years with a disease duration of 9.6 ± 8.1 years (<1 to 50 years).

**Results:**

Overall, 18.4% patients had HbA1c levels <7.0%, and 47.5% patients had HbA1c levels ≥ 9%. HbA1c levels were associated with lower economic status, female gender, age and the daily frequency of self-blood glucose monitoring (SBGM) but not with insulin regimen and geographic region. Hypertension was more frequent in the mid-west (32%) and north/northeast (25%) than in the southeast (19%) and south (17%) regions (p<0.001). More patients from the southeast region achieved LDL cholesterol goals and were treated with statins (p<0.001). Fewer patients from the north/northeast and mid-west regions were screened for retinopathy and nephropathy, compared with patients from the south and southeast. Patients from the south/southeast regions had more intensive insulin regimens than patients from the north/northeast and mid-west regions (p<0.001). The most common insulin therapy combination was intermediate-acting with regular human insulin, mainly in the north/northeast region (p<0.001). The combination of insulin glargine with lispro and glulisine was more frequently used in the mid-west region (p<0.001). Patients from the north/northeast region were younger, non-Caucasian, from lower economic status, used less continuous subcutaneous insulin infusion, performed less SBGM and were less overweight/obese (p<0.001).

**Conclusions:**

A majority of patients, mainly in the north/northeast and mid-west regions, did not meet metabolic control goals and were not screened for diabetes-related chronic complications. These results should guide governmental health policy decisions, specific to each geographic region, to improve diabetes care and decrease the negative impact diabetes has on the public health system.

## Background

Type 1 diabetes mellitus (T1D) is a chronic autoimmune disease and both genetic and environmental factors have an important role in its onset. However, despite a large amount of research that has been conducted in recent decades, the causal factors of this disease are still unknown [1]. According to the World Health Organization (WHO), the incidence of T1D is increasing worldwide [2]. This fact has been observed in developed [3] and developing countries, including Brazil [4]. T1D carries a great risk of morbidity and mortality due to the microvascular and macrovascular complications that can lead to a lower quality of life and life expectancy [5]. Currently, these complications can be postponed by achieving adequate glycemic control, as demonstrated by the Diabetes Control and Complications Trial, the Epidemiology of Diabetes Interventions and Complications Trial and the long-term follow-up study of the Diabetes Control and Complications Trial [6,7]. However, many barriers to achieve adequate glycemic control have been observed in observational studies, including lack of family support, fear of hypoglycemia, difficulties in the day-to-day management of T1D (mainly frequent self-blood glucose monitoring (SBGM) for insulin dose adjustments), diet, exercise and economic status [8]. Considering the complexity and cost of following the recommended guidelines for clinical management of T1D, economic status represents an important issue in developing countries [9].

Brazil is the fifth largest country in the world. It has a tropical climate and comprises 20.8% of American territory and 47.7% of South American territory and an estimated population of 191.8 million people. This results in a demographic density of 22.5 inhabitants/km^2^ according to the last population census conducted in 2009 by the Brazilian Institute of Geography and Statistics (IBGE) [9]. The self-reported ethnicity is mainly composed of Caucasians (54%), followed by Mullatos (44.25%), Afro-Brazilians (6.9%) and Natives (less than 1%). The annual per capita income in 2011 was estimated to be US $12,144 with an uneven distribution across the geographic regions of the country [10]. Currently, it is estimated that up to 9.7% of the Brazilian population is illiterate, although there is great variation, ranging from 4.26 to 12.21% across the different geographic regions [9]. Considering the above-mentioned data, as well as the fact that few national data about the clinical care of T1D across the geographic regions exist thus far, the Brazilian Type 1 Diabetes Study Group (BrazDiab1SG) was organized in 2008. The BrazDiab1SG is a survey that analyzes the demographic, clinical and socioeconomic data of T1D patients who have attended public clinics across Brazil.

The present study aimed to determine the characteristics of clinical care offered to patients with T1D in Brazil, including the degree of glycemic control, presence of cardiovascular (CV) risk factors and frequency of screening for chronic T1D-related complications using the American Diabetes Association’s (ADA) guidelines. In addition, the present study aimed to evaluate health care practices, disease management and the frequency that the recommended treatment goals were met.

## Methods

This study was an observational, cross-sectional, multicenter study conducted between December 2008 and December 2010 in 28 secondary and tertiary care public clinics located in 20 cities in four Brazilian geographic regions (north/northeast, mid-west, southeast and south). Detailed methods have been described elsewhere [11]. Briefly, all patients received health care from the National Brazilian Health Care System (NBHCS). To be eligible, the participating centers had to have a diabetes clinic with at least one endocrinologist. Each clinic provided data from at least 50 consecutive outpatients with an initial diagnosis of T1D who regularly attended the clinic. The inclusion criteria included a diagnosis of T1D by a physician based on typical clinical presentation, including a variable degree of weight loss, polyuria, polydipsia and polyphagia, in addition to the need to use insulin continuously since the diagnosis without interruption. All patients were diagnosed between 1960 and 2010.

Demographic data, educational data and economic status were also obtained. Patients with diabetes for less than five years in duration, were not included in the analysis of diabetic complications (n=1,160; 32.3%). As listed in Appendix 1, each local center’s ethics committee approved the study. The Brazilian Diabetes Society coordinated the study by monitoring and reviewing all study-related documents and approving all amendments and publications. The standardized form used for data collection was reviewed by leading diabetologists in Brazil before the final approval.

The following variables were assessed through an interview during a clinical visit: current age, age at diagnosis, duration of diabetes (y), height (m), weight (kg), blood pressure (systolic and diastolic in mmHg), diabetes treatment modalities, comorbidities, frequency of SBGM and smoking status. HbA1c, fasting plasma glucose (FPG), total cholesterol, LDL cholesterol, HDL cholesterol and triglycerides levels recorded during the last visit to the clinic were obtained from the medical records. In addition, it was also documented whether retinopathy screening by fundoscopy, nephropathy screening by microalbuminuria or a foot examination had been conducted within one year of the study assessment in patients who had been diagnosed with diabetes for longer than five years [12].

The following ADA goals for good metabolic and clinical control [12] were adopted by the BrazDiab1SG: HbA1c < 7.5% for T1D patients 13 to 19 years of age; HbA1c <8% for T1D patients 6 to 12 years of age; HbA1c > 7.5% and < 8.5 % for T1D patients aged less than 6 years old; HbA1c < 7% for adult T1D patients; systolic blood pressure (sBP) < 130 mmHg; diastolic blood pressure (dBP) < 80 mmHg; body mass index (BMI) < 25 kg/m^2^; FPG < 130 mg/dl (7.2 mmol/l); total cholesterol < 200 mg/dl (5.2 mmol/l); HDL cholesterol > 40 mg/dl for men (1.1 mmol/l) and > 50 mg/dl (1.3 mmol/l) for women; LDL cholesterol < 100 mg/dl (2.6 mmol/l); non-HDL cholesterol < 130 mg/dl (3.30 mmol/l); and triglycerides < 150 mg/dl/l (1.7 mmol/l).

In adults, hypertension was defined as a measurement of sBP ≥ 140 mmHg and/or dBP ≥ 90 mmHg during the last visit [12], the use of an anti-hypertensive drug or self-reported identification. In children and adolescents, hypertension was defined as a sBP or dBP ≥ the 95th percentile for age, sex and height [13]. In adults, overweight was defined as having a BMI ≥ 25 kg/m^2^, and obesity was defined as having a BMI ≥ 30 kg/m^2^[14]. In children and adolescents, overweight was defined as having a BMI ≥ the 85th percentile for age and gender, and obesity was defined as having a BMI ≥ the 95th percentile for age and gender [14].

HbA1c values and the methods used for its measurement were collected from medical charts. In 3,367 patients (93.7%), HbA1c levels were measured using methods certified by the National Glycohemoglobin Standardization Program (NGSP), including high-performance liquid chromatography in 1,766 patients (51.3%) and turbidimetry in 1,601 patients (46.6%). HbA1c levels determined using methods not certified by the NGSP, missing data and HbA1c levels determined more than one year before the study assessment were excluded from the analysis of glycemic control (n=494; 13.8%). FPG, triglycerides, HDL and total cholesterol were measured using enzymatic techniques. LDL levels were calculated using Friedewald’s equation [15]. BMI (kg/m^2^) was determined by dividing the weight (kg) by the square of the height (m). A current smoking habit was defined as a patient smoking more than one cigarette per day at the time of the interview. The classification of patients regarding age was as follows: patients <13 years old were considered children; patients ≥13 years were deemed as adolescents; and patients >18 years were considered to be adults [12]. Written informed consent for the study was obtained from all the patients or from the patients’ parents, when necessary.

### Statistical analysis

A detailed description of the study sample calculation has been previously described [11]. Briefly, the study sample aimed to represent the distribution of T1D cases across the various geographic regions of Brazil. The proportion of cases in each region was estimated using the overall population distribution reported in the Population Census conducted in 2000 by the IBGE to be 38.8, 31.7, 23.0 and 6.6% in the southeast, north/northeast, south and mid-west regions, respectively [16]. These numbers were combined with national estimates of the prevalence of diabetes derived from a survey performed in 1988 to determine the minimum number of patients to be studied in each region [17]. Concerning the recruitment, each region of the country enrolled > 95% of its estimated number of T1D patients. Economic status was defined according to the Brazilian Economic Classification Criteria [18]. This classification also takes into account education level, which was categorized as illiterate/incomplete primary education, complete primary education/incomplete middle school education, complete middle school education/incomplete high school education, complete high school education /some college education or complete college education. For this analysis, the following classes of economic status were considered: high, middle, low and very low income classes [18].

The data are presented as the means (± SD) for continuous variables and as counts (relative frequencies) for discrete variables. Comparisons between genders were performed using independent two-sided t-tests or ANOVA for continuous variables and two-sided z-tests for discrete variables with a normal approximation to the binomial distribution. Due to the number of variables tested sequentially, when ANOVAs were used, p-values were adjusted using the Sidák correction procedure to control for type I errors and when z-tests were used, p values < 0.005 were considered significant. For the other analyses, a two-sided p-value less than 0.05 was considered significant.

Pearson’s correlation coefficient was calculated when appropriate. Variables that were not normally distributed were log transformed. Stepwise multiple regression was performed with HbA1c levels as dependent variable and as independent variables, we analyzed the data with the Person correlation coefficient of <0.1. A multiple logistic regression (Forward-Wald) was performed with hypertension (yes/no) as the dependent variable. The following independent variables were included: ethnicity (Caucasian or non-Caucasian based on medical charts or self-reported), age, BMI, geographic region, gender, urine albumin concentration and economic status. For this analysis, the Nagelkerke R-squared value was also calculated. All of the analyses were performed using SPSS version 16.0 (Statistical Package of Social Sciences, Chicago, Illinois). Odds ratios with 95% confidence intervals (CI) were expressed when appropriate.

## Results

### Demographics, socioeconomic status, level of care and treatment modalities

The distribution of the study population across the geographic regions of the country and the clinical and demographic data are shown in Table 1. The majority of the patients were diagnosed before the age of 15 (n=2,574; 71.7%). The durations of diabetes in the patients were as follows: <1 to 5 y, n=1,160 (32.3%); 5 to 10 y, n=966 (26.9%); 10 to 15 y, n=642 (17.9%); 15 to 20 y, n=404 (11.3%); and ≥20 y, n=419 (11.7%). Overall, 995 (27.7%) patients were treated at the secondary care level, and 2,596 (72.3%) patients were treated at the tertiary care level. More patients from the north/northeast and mid-west regions attended secondary care centers compared to patients from the southeast and south regions (60.9, 73.1, 7.1 and 5.5% for north/northeast, mid-west, southeast and south, respectively; p<0.001). The average follow-up time in the participating centers was 6.1 ± 5.8 y.

**Table 1:** Clinical and demographic data of the study population according to geographic region

**Variable**	**Southeast**	**South**	**North/ Northeast**	**Mid-west**	**P Value**
**N(%)**	1,424 (39.7)	820 (22.8)	1,113 (31.0)	234 (6.5)	
**Gender, F (%)**	821 (57.7)	461 (56.2)	593 (53.3)	135 (57.7)	0.21
**Age, y**	21.6 ± 12.4	22.3 ± 12.2	19.9 ± 10.1^†^	21.2 ± 11.9	<0.001
**Age, y (%)**					0.19
0–5.9	57 (4)	18 (2.2)	41 (3.7)	14 (6.0)	
6–12.9	279 (19.6)	148 (18.0)	239 (21.5)	56 (23.9)	
13–18.9	370 (26.0)	225 (27.4)	296 (26.6)	40 (17.1)	
≥19.0	719 (50.4)	429 (52.3)	537 (48.2)	124 (53.0)	
**Age at diagnosis, y (%)**					<0.001
0–4.9	318 (22.3)	133 (16.2)	169 (15.2)	47 (20.1)	
5–9.9	401 (28.2)	227 (27.7)	280 (25.2)	53 (22.6)	
10–14.9	370 (26.0)	243 (29.6)	285 (25.6)	43 (18.4)	
15-19.9	160 (11.2)	103 (12.6)	186 (16.7)	43 (18.4)	
20–29.9	121 (8.5)	82 (10.0)	160 (14.4)	41 (17.5)	
≥30	54 (3.8)	32 (3.9)	33 (3.0)	7 (3.0)	
**Ethnicity, n (%)**					<0.001
Caucasian	830 (58.9)	716 (87.3)	383 (34.4)	111 (47.4)	
Non-Caucasian*	585 (41.1)	104 (12.7)	730 (65.6)	123 (52.6)	
Economic status**					<0.001
High	98 (7.2)	82 (10.2)	36 (3.4)	24 (10.9)	
Medium	361(26.4)	248 (30.9)	101 (9.4)	63 (28.6)	
Low	496 (36.3)	311 (38.1)	302 (28.2)	68 (30.9)	
Very low	412 (30.1)	161 (20.1)	633 (59.0)	65 (29.5)	
Years of Study	9.7 ± 4.5	9.5 ± 3.9	9.9 ± 4.5	10.5 ± 5.2^†^	0.01
**Duration of diabetes, y**	10.7 ± 8.8	10.6 ± 8.4	7.6 ± 6.4^††^	8.6 ± 7.5^††^	<0.001
**Treatment of diabetes n(%)**					
Insulin ***					<0.001
Intermediate or long acting	157 (11)	49 (6.0)	291 (26.2)	62 (26.5)	
CSII****	23 (1.6)	10 (1.2)	2 (0.2)	4 (1.7)	
Intermediate/long plus short acting	1,241 (87.3)	761 (92.8)	819 (73.7)	168 (71.8)	
Short acting shots ≥ 3 /day	832 (64.7)	570 (73.0)	408 (48.7)	103 (50.9)	<0.001
SBGM, yes (%)	1,331(93.5)	763 (93.0)	853 (76.6) ^†††^	221 (94.4)	
Specialist visits in the prior, y	1,424(99.0)	819 (99.8)	1,097 (98.5)	226 (96.6)	0.3
Level of care n (%)					
Secondary	101(7.1)	45 (5.5)	678 (60.9)	171(73.1)	<0.001
Tertiary	1,323(92.9)	775(94.5)	435 (39.1)^#^	63(26.9)^#^	

The data are presented as the means (SD) and n (%); y = years; f = female, SBGM = self-blood glucose monitoring.^†^ p<0.01 *vs.* each other region; ^††^P<0.05 *vs.* each other region; ^†††^ p<0.001 *vs. each* other region ^#^ P< 0.01 *vs.* south and southeast regions.

* African-Brazilians, Mulattos, Asians, Native Aborigines.

** Data not available from 130 (3.6%) patients *** Data not available from 4 (0.001%) patients.

****CSII: continuous subcutaneous insulin infusion.

For comparison between continuous variables ANOVA with Sidak correction were used; For comparison between discrete variables two-sided z-tests with correction was used.

There were differences in age, age at diagnosis, ethnicity, economic status, care level and duration of diabetes across the regions of the country. Patients from the north/northeast region were younger, non-Caucasian and from very low or low economic status, compared to patients from the other regions of the country (Table 1).

Diabetes treatment was also different across the regions. Fewer patients from the north/northeast region used continuous subcutaneous insulin infusion (CSII) and performed SBGM daily (p<0.05). Overall, 559 patients (15.6%) used only intermediate-acting human insulin (NPH) or long-acting insulin (glargine or detemir); there was a significant difference in the use of either intermediate-acting human insulin or long-acting-insulin (glargine or detemir) across the geographic regions (p<0.001). The most frequently found modality of insulin monotherapy was the use of intermediate-acting human insulin (NPH) (Table 1). More patients from the south and mid-west regions used only insulin glargine or detemir compared to patients from the southeast or northeast/north regions (p<0.001) (Table 1).

More patients from the south/southeast region were treated with intermediate-acting/long-acting plus short-acting insulin and used three or more daily injections of short-acting insulin, compared to patients from the north/northeast and mid-west regions (Table 1).

Insulin therapy combinations were used by 2,989 (83.2%) patients, and there were large differences among the types of combinations used across the country (p<0.001). The most frequent combination of insulin therapy for all the geographic regions was intermediate-acting human insulin (NPH) with human regular insulin. This type of combination was more frequent in the north/northeast region compared to the other regions (p<0.001). The combinations of insulin glargine with insulin lispro and insulin glargine with insulin glulisine were more frequently used by patients from the mid-west region (p<0.001). These data are shown in Table 2.

**Table 2:** Types of insulin therapy according to geographic region

	Insulin Monotherapy *			
**Type of insulin monotherapy**	**Southeast**	**South**	**Northeast/North**	**Mid-west**
NPH	152 (96.8)	41 (83.7)	275 (94.5)	54 (87.1)
Glargine or Detemir	5 (3.2)	8 (16.3)	16 (5.5)	8 (12.9)
	Combined Insulin Therapy**			
**Type of combination of insulin therapy**	**Southeast**	**South**	**Northeast/North**	**Mid-west**
NPH plus Regular	573 (46.2)	357 (46.9)	677 (82.7)	79 (47.0)
NPH plus Lispro	271 (21.8)	194 (25.5)	12 (1.5)	11 (6.5)
NPH plus Aspart	91 (7.3)	38 (5.0)	13 (1.6)	4 (2.4)
NPH plus Glulisine	6 (0.5)	4 (0.5)	7 (0.9)	5 (3)
Glargine plus regular	16 (1.3)	12 (1.6)	10 (1.2)	1 (0.6)
Glargine plus Lispro	158 (12.7)	71 (9.3)	25 (3.1)	48 (28.6)
Glargine plus Aspart	57 (4.6)	67 (8.8)	32 (3.9)	6 (3.6)
Glargine plus Glulisine	2 (0.2)	3 (0.4)	3 (0.4)	13 (7.7)
Detemir plus regular	4 (0.3)	-	5 (0.6)	-
Detemir plus Lispro	25 (2)	3 (0.4)	9 (1.1)	1 (0.6)
Detemir plus Aspart	37 (3)	12 (1.6)	24 (2.9)	-
Detemir plus Glulisine	1 (0.1)	-	2 (0.2)	-

The data are presented as n (%); * /** p<0.001 for comparisons among the groups.

### Glycemic control

Clinical and laboratory data, as well as the proportions of patients who underwent clinical and biochemical evaluations showing that they achieved the ADA criteria for good metabolic control in the various geographic regions are shown in Table 3. Overall, more patients from the north/northeast (n=358; 32.2%) and mid-west (n=38; 16.2%) regions than patients from the southeast (n=83; 5.8%) and south (n=15; 1.8%) regions had their HbA1c levels excluded due to the following reasons: missing data; HbA1c levels were determined using methods not certified by the NGSP; or HbA1c levels were determined more than one year before the study assessment. The frequencies of HbA1c level determinations per year were also different across the country (2.8 ± 1.4, 3.0 ± 1.0, 2.0 ± 1.2, and 1.9 ± 1.0/year for the southeast, south, north/northeast and mid-west, respectively; p<0.001).

**Table 3:** Clinical and laboratory data and screening for chronic complications in the study population according to geographic region

	**Southeast**	**South**	**North/Northeast**	**Mid-west**	**P Value**
**N(%)**	1,424(39.7)	820 (22.8)	1,113(31.0)	234 (6.5)	
***Glycemic Control***					
HbA1c					
Frequency of Complete Records n (%)	1,356 (95.2)	809 (98.7)	978(87.9)	210 (89.7)	<0.001
Mean (SD)	9.1 ± 2.3	9.4 ± 2.1^#^	9.4 ± 2.6^#^	9.1 ± 2.6	0.002
Patients at Goal	276 (20.6)	98 (12.2)*	154 (20.4)	42 (21.4)	<0.001
FPG					
Frequency of Complete Records n (%)	1,251 (87.9)	803 (97.9)	1,016 (91.3)	187 (79.9)	
Mean (SD)	187.3 (108.4)^#^	174.4 (94.4)	185.3 ± 108.2^#^	179.0 ± 102.60	<0.001
Patients at Goal	332 (26.6)	293 (36.5)	301 (29.6)	53 (28.3)	<0.001
***Cardiovascular Risk Factors***					
BMI	1,419 (99.6)	819 (99.9)	1,104 (99.2)	226 (96.6)	
Frequency of Complete Records n (%)					
Mean (SD)	21.9 ± 4.4	22.2 ±3.3*	20.9 ± 4.2	21.7 ± 4.6	0.01
Patients at Goal	942 (66.4)	547 (66.8)	823 (74.5)*	150 (66.4)	0.009
***sBP***					
Frequency of Complete Records n (%)	1,393 (97.8)	811 (98.9)	1,040 (93.4)	193 (82.5)	
Mean (SD)	111.4 ± 16.0	111.7 ± 17.9	109.1 ± 17.4*	115.8 (19.3)	<0.001
Patients at Goal	1,159 (83.2)	667 (82.2)	893 (85.9)	148 (76.7)	0.009
***dBP***					
Frequency of Complete Records n (%)	1,392 (97.8)	811 (98.9)	1,032 (92.7)	191 (81.6)	
Mean (SD) mmHg	71.4 ± 11.02	71.4 ± 11.7	70.9 ± 12.2	71.8 ± 12.2	0.31
Patients at Goal	890 (63.9)	523 (64.5)	709 (68.7)	137 (71.7)*	0.003
*Triglycerides (mg/dl)*					
Frequency of Complete Records n (%)	1,262 (88.6)	496 (60.5)	853 (76.6)	181 (77.4)	
Mean (SD)	87.7 ± 69.1	95.3 ± 72.1	103.5 ± 76.9	91.6 ± 58.1	0.006
Patients at Goal	986 (78.1)	407 (82.1)	589 (69.1)*	145 (80.1)	0.001
*HDL (Cholesterol)*					
Frequency of Complete Records n (%)	1,227 (86.2)	487 (59.4)	838 (75.3)	180 (76.9)	
Mean (SD)	53.3 ± 14.8*	53.9 ± 15.2***	50.1 ± 14.1	52.0 ± 12.1	0.01
Patients at Goal n (%)	984 (80.2)****	369 (75.8)	604 (72.1)	141 (78.3)	0.004
*LDL Cholesterol*					
*Frequency of Complete Records n (%)*	1,218 (85.5)	483 (58.9)	821 (73.8)	179 (76.5)	
Mean (SD)	96.9 ± 33.1***	102.6 ± 31.3	102.9 ± 35.7	102.1 ± 28.3	<0.002
Patients at Goal n (%)	730 (59.9)*	241 (49.9)	422 (51.4)	89 (49.7)	<0.001
*Non-HDL Cholesterol*	1,227 (86.2)	487(59.4)	835 (75)	180 (76.9)	
*Frequency of Complete Records n (%)*					
Mean (SD)	113.7 ± 39.1 ^###^	121.6 ± 37.5*	122.0 ± 43.1*	119.5 ± 32.7	0.001
Patients at Goal	924 (75.3)****	322 (66.1)	529 (63.4)	123 (68.3)	<0.001
Smoking Status (y), N (%)	56 (4.1)	60 (7.5)	24 (2.2)	10 (4.5)	0.000
***Chronic Complications***					
*Feet examination*					
Yes, n(%)	1,071 (75.2)*	465 (56.7)	588 (52.8)	128 (54.7)	<0.001
*Fundoscopy y, n(%)*					
Yes,n (%)	714 (50.1)^+^	462 (56.3)*	427 (38.4)	80 (34.2)	<0.001
*Urine Albumin*					
Yes, n (%)	661 (46.4)^+^	484 (59)*	361 (32.4)	72 (30.8)	<0.001

Data are presented as the means (SD) and n (%); y = yes * P<0.001 *vs.* each other region; *** p<0.002 *vs.* each other region; **** p<0.01 *vs.* each other region; ### p<0.005 *vs.* each other region; + p<0.01 *vs.* north/northeast and mid–west regions For comparison between continuous variables ANOVA with Sidak correction were used; For comparison between discrete variables two-sided z-tests with correction was used.

Overall, 570 (18.4%) patients achieved the ADA criteria for good metabolic control, with the majority of these patients (48.5%) being from the southeast region. Of the entire study population, 1,472 (47.5%) patients had HbA1c levels ≥ 9%.

When stepwise multiple regression analysis was applied with HbA1c level as the dependent variable and the independent variables being the geographic region of the country, duration of diabetes, insulin regimen, age, economic status, daily frequency of SBGM and gender, it was shown that low/very low economic status, (r=0.10, r^2^=0.01, and B=0.22; p < 0.001), daily frequency of SBGM (r=0.14, r^2^=0.02, and B=−0.13; p = 0.001), female gender (r=0.16, r^2^=0.02, and B=0.40; p < 0.001) and age (r=0.17, r^2^=0.03, and B=−0.013; p< 0.001) were associated with HbA1c levels. Associations with insulin regimen, region of the country and duration of diabetes did not reach statistical significance.

### Cardiovascular risk factors and specific therapy

Clinical and laboratory data, as well as the proportions of patients who underwent clinical and biochemical evaluations showing that they achieved the ADA criteria for CV risk factors according to the geographic regions are shown in Table 3.

Overweight and obesity were observed in 820 (22.8%) and 286 (8.0%) patients, respectively. More patients from the north/northeast region achieved the healthy target BMI (p<0.001) than patients from other regions of the country. A current smoking status was reported by 150 (4.2%) patients and was more frequent among patients from the South region than among patients from other regions of the country (p<0.001).

Hypertension was observed in 689 (19.2%) patients, and the frequency of hypertension was also different among the regions of the country (32.0, 25.0, 19.0 and 17.0% for the mid-west, north/northeast, southeast and south, respectively, p<001). Among all of the patients with hypertension, 333 (48.1%) underwent treatment. Among the hypertensive patients, 321 (47.1%) and 240 (35.2%) were within the targets for sBP and dBP, respectively. Overall, 76 (22.9%) of the patients with hypertension who were using anti-hypertensive agents were within the targets for both sBP and dBP.

Five hundred thirty-four patients (14.9%) used anti-hypertensive agents, and the use of anti-hypertensive agents was related to the region of the country (44.9, 28.1, 20.4 and 6.6% for the southeast, south, north/northeast and mid-west, respectively; p<0.001). The most frequently used anti-hypertensive agents were angiotensin-converting-enzyme (ACE) inhibitors, which were used by 335 (57.2%) patients.

When multivariate logistic analysis was applied, the odds of having hypertension was associated with age (1.06; 95%CI of 1.05-1.076; p<0.001), BMI (1.13; 95%CI of 1.09-1.17; p<0.001), albumin excretion rate (1.002; 95%CI of 1.001-1.003; p<0.001) and male gender (1.35; 95%CI of 1.02-1.80; p<0.001). Caucasian ethnicity was associated with a lower odds ratio of having hypertension (0.68; 95% CI of 0.51-0.91; p=0.01). This model explained 25.3% of the probability for a given patient to be hypertensive.

Overall, 1,785 (49.7%) patients had some type of dyslipidemia. Considering the goals for lipid levels, fewer patients from the north/northeast region achieved the target for triglycerides and HDL cholesterol levels than patients from other regions of the country (p=0.001). More patients from the southeast region achieved the target for LDL cholesterol levels than patients from the other regions (p<0.001).

Statins were used by 284 (7.9%) patients, and 112 of these (42.1%) were within the target for LDL cholesterol levels. Overall, 1,065 (43.7%) patients had LDL cholesterol levels above the target levels and were not receiving treatment. More patients from the Southeast region were using statins than patients from the other regions (49.3, 28.5, 17.6 and 4.6% for patients from the southeast, south, north/northeast and mid-west, respectively; p<0.001).

### Screening for microvascular complications

Screening for microvascular complications was different across the regions of the country (Table 3). Overall, 25 to 70% of the patients had not been screened for diabetes-related complications in the previous year. More patients from the north/northeast and mid-west regions than patients from the southeast and south regions had not been screened for diabetic chronic complications in the previous year. More patients from the south region had fundoscopies and urine albumin excretion rate evaluations than patients from the other regions (p<0.001). Furthermore, up to 25% of the records did not have any information about screening for diabetes-related complications (data not shown) in the prior year.

Among the patients that had been screened for chronic diabetes-related complications, 630 (25.9%) had been screened for coronary artery disease. More patients from the southeast region had been screened than patients from the south, north/northeast and mid-west regions (45.9, 22.4, 27.3 and 4.4% for the southeast, south, north/northeast and mid-west, respectively). The most frequently performed tests in this group were electrocardiography (18.6%) and treadmill stress tests (6.7%).

## Discussion

The BrazDiab1SG is a survey that analyzes the demographic, clinical and socioeconomic data of T1D patients receiving treatment in secondary and tertiary care public clinics in Brazil.

In the present study, great variability was found in the proportion of patients screened for diabetes-related complications and in the proportion of patients reaching their targets depending on the variables evaluated and on the geographic region of the country. The southeast and south regions were quite similar regarding patient’s demographic and economic characteristics but with an important difference from the patients from the north/northeast and mid-west. Overall, more patients from the north/northeast attended secondary care level centers, tended to be younger, tended to be non-Caucasian and tended to be from a lower economic status.

For most patients, blood pressure levels were within the goals. However, glycemic control was unsatisfactory in the majority of the study patients. Being overweight was an important issue in all regions of the country and was observed in one-third of the study patients. Additionally, approximately half of the patients were not screened for diabetic complications in the previous year. It is important to mention that all of the patients were treated by an endocrinologist at secondary and tertiary care clinics.

The treatment of diabetes in Brazil is guided by the Brazilian Diabetes Society , whose recommendations are essentially the same as those of the ADA. Considering that diabetes treatment in public clinics is financed by the National Brazilian Health Care System, the present data showed that factors other than medical recommendations likely interfere with diabetes care in Brazil, especially social and economic factors. The latter fact may be associated with the low or very low economic status that was found in up to 87% of the studied patients, mainly in the north/northeast region of the country. Notably, economic status in Brazil also takes into account educational level. In the last Brazilian population census [9], the north/northeast region presented a higher number of illiterates (12.2%) than the national average (9.7%) and the proportion observed in the southeast (4.45%), south (4.26%), and mid-west (6%). Considering the existing complexity of T1D management, the above-mentioned facts may have influenced the lower use of CSII, SBGM and intermediate-acting/long-acting plus short-acting insulin by the north/northeast region. Although patients from the north/northeast region had a lower duration of diabetes, it is important to emphasize that no difference was observed concerning the number of visits to a specialist in the prior year among all of the geographic regions of the country.

The present study found great heterogeneity in the type of human intermediate-acting/long-acting plus short-acting insulin used by the patients across the Brazilian geographic regions. Although the most frequent type of association in all regions was human intermediate-acting insulin NPH with regular human insulin, the combination of insulin glargine with insulin lispro or glulisine was more frequently used in the mid-west region. No clear explanation exists for this trend, but it may be explained by the local health policy responsible for the acquaintance of insulin. Although diabetes treatment in public clinics is financed by the National Brazilian Health Care System, it is important to emphasize that each city’s health bureau acts independently from the federal government and has its own rules concerning the choice of what type of insulin they are going to purchase and furnish to their diabetic population.

Approximately 13.8% of the HbA1c determinations, mainly in the north/northeast and mid-west regions, were excluded due to the following reasons: the methods used were not certified by the NGSP; existence of missing data; or the HbA1c level was determined more than one year before the study assessment. Moreover, among those patients who had undergone HbA1c measurements in the prior year to the study, the average number of measurements was also different across the geographic regions, with a lower number performed in the north/northeast and mid-west regions. In general, patients from the southeast and south regions performed a similar number of HbA1c determination to that proposed by ADA guidelines [12] and the Brazilian Diabetes Society recommendations [19] but varied widely from one to ten per year. This range indicates that there is no agreement in Brazil regarding the number of HbA1c measurements routinely performed to monitor the treatment of a patient with T1D. Other factors related to the physician’s interpretation of the test might explain these discrepancies.

Although most patients had complex therapeutic regimens and performed SBGM, more than 40% of the patients had HbA1c levels greater than 9%, thereby indicating poor glycemic control. Although the north/northeast and south regions had the highest average HbA1c levels, the lowest proportion of patients reaching the goal of HbA1c was observed in the south region. However, it is important to emphasize that in the multivariate analysis (after correcting for age, economic status, gender and daily frequency of SBGM) neither the geographic region of the country nor the type of insulin regimen that was used reached statistical significance. The latter fact must be analyzed in the context of the high costs of CSII and long-acting insulin analogues (glargine and detemir). Although the present study was not designed to evaluate the cost-effectiveness of both types of treatment, the data could add new insights for reforming the guidelines of the National Brazilian Health Care System concerning the treatment of T1D in Brazil. Thus far, the majority of the studies that have addressed this issue have found that both types of treatment are mainly associated with an improvement in the occurrence of severe hypoglycemia but not with improved glycemic control, thereby showing that a gap exists between the large amounts of money spent with the treatment of T1D and the final outcomes in terms of glycemic and CV risk factors control [20-24]. However, the overall difficulty in achieving glycemic control in T1D patients through routine care is described in many observational studies worldwide [17-19,25-28].

Currently, the therapeutic and clinical management of weight, cholesterol and blood pressure is of great importance for delaying or preventing diabetes-related microvascular and macrovascular complications. Although most of the studied patients achieved the sBP, dBP and HDL cholesterol goals, 30 to 50% did not reach the goals for triglycerides, LDL cholesterol and non-HDL cholesterol levels, which was similar to the data found in other observational studies [7,18,28-30]. Although there is a slight prevalence of patients that have reached BMI levels resembling Brazilian overweight/obesity statistics [31], the present results suggested an additional major health issue in T1D patients in all geographic regions is overweight or obese patients, which was similar to results found in other populations with T1D [7,18,32,33] and in patients with T2D in Brazil [9].

Although the guidelines recommend aggressive dyslipidemia and hypertension treatments in T1D patients, despite the presence of high BP and LDL cholesterol, up to 50% of the studied patients were not receiving treatment for both clinical conditions during this study. Similar results have also been described in Sweden [18]. Hypertension was observed in 19.2% of the patients with a higher proportion in patients from the mid-west and north/northeast regions than patients from the southeast and south regions. Until recently, no data were available concerning hypertension prevalence in T1D Brazilian patients. Overall, almost half of the studied patients had some type of dyslipidemia, but only 7.9% of these patients were using statins for dyslipidemia. Of these patients, fewer than 50% were at LDL cholesterol goals. More patients from the southeast reached the target for LDL cholesterol than patients from the other regions, which may have been due to the more regular use of statins than patients from the other geographic regions. To the best of our knowledge, this is the first study to address the abovementioned comorbidities in T1D in a multicenter study in Brazil.

The Pittsburgh Epidemiology of Diabetes Complications Study used different targets for blood pressure and LDL cholesterol, and it demonstrated small improvements in hypertension and dyslipidemia control primarily in younger groups of T1D patients over a 10-year follow-up period [29]. With regard to T1D patients who were above their goal, one study at academic medical centers observed a low rate of medication management [8]. Considering overweight, obesity, hypertension and dyslipidemia as CV risk factors, it can be concluded that the young patients in the present study represent a high-risk group for microvascular and macrovascular complications of diabetes, as described in other studies [30,32,33]. Although a high number of current smokers were noted in the south region in the present study, there were fewer T1D patients who were current smokers than previously reported in Europe [18] and USA [34].

Despite that almost one-third of the patients did not fulfill the criteria for screening for diabetic complications, up to 60% of the patients conforming to the inclusion criteria had not been screened for diabetic chronic complications in the previous year. In general, the screening for diabetic chronic complications was documented more in the south region, followed by the southeast region. Fundoscopy and urine albumin testing were not documented in up 60% of the patients. Notably, urine albumin evaluation is now regularly performed in 75% of the public hospitals included in the present study. Moreover, the simplest screening for diabetes-related complications, which is feet examination, was not routinely performed in up to 45% of the patients.

The principal strength of the large sample groups in the present study is that the included cases are representative of the distribution of T1D in the diverse, young Brazilian population. Moreover, the epidemiological information obtained from this study is important for guiding governmental health policy decisions aimed at improving diabetes care in Brazil, according to each geographic region of the country.

Several limitations of the present study must be addressed. One limitation is the sample characteristics. Similar to other studies, a clinical definition of T1D assigned by physicians that was applicable to all patients was used. However, as autoantibodies and C-peptide levels were not measured, some patients with other types of diabetes may have been included. Nevertheless, it is important to emphasize that 96.5% of the patients were diagnosed before 30 years of age, which reinforces the high probability that they most likely had T1D. All patients were followed in a public center by a specialist and lived in large cities. Patients who rely on private clinics, primary care facilities and live in rural areas may not have been included. However, this group of T1D patients is considered to be the minority of those receiving treatment in Brazil. Another limitation is the lack of standardization for evaluating HbA1c levels. Although two different methods to determine HbA1c levels were used across the country, different upper limits of normality for the same method may be present. This variation may have also influenced the results of the present study. However, this variation in methods is still an unsolved problem in Brazil. Moreover, the consideration of self-reported hypertension as a criterion of the presence of the comorbidity may have led to misdiagnosis and lower reliability in determining the real prevalence of hypertension.

## Conclusions

In conclusion, sufficient screening for diabetic complications and the target levels for glycemic control, blood pressure and lipid levels are difficult to achieve in patients with T1D. Multiple CV risk factors were found in most patients. The quality of diabetes care must be substantially improved in Brazil. Among T1D patients studied in the BrazDiab1SG, large discrepancies were found in clinical care across the different Brazilian geographic regions. With few exceptions regarding the differences found, this study reinforces the need for a uniform use of the guideline-recommended therapies, either by the Brazilian Diabetes Society or by the ADA, to improve the quality of diabetes care in Brazil.

Future larger Brazilian prospective studies are warranted to evaluate the cost-effectiveness of more expensive treatment modalities, such as CSII, the use of long-acting insulin analogues (glargine and detemir), as well as the costless distribution of strips for SBGM in the context of the Brazilian healthcare system. The large amount of money that has been spent with these treatment modalities is not showing an expected positive feed-back in terms of glycemic control and the improvement of cardiovascular risk factors in patients with T1D in Brazil.

## Appendix 1

**Universidade Estado Rio de Janeiro**: Roberta Cobas*, Alessandra Matheus, Lucianne Tannus; **Universidade Federal Rio de Janeiro**: Lenita Zajdenverg*, Melanie Rodacki; **Hospital Geral de Bonsucesso**: Neuza Braga Campos de Araújo*, Marilena de Menezes Cordeiro; **Hospital Universitário Clementino Fraga Filho – IPPMG**: Dr. Jorge Luiz Luescher*; Renata Szundy Berardo; **Serviço de Diabetes da Disciplina de Endocrinologia e Metabologia do Hospital das Clínicas da Universidade de São Paulo**: Marcia Nery*; Catarina Cani; Maria do Carmo Arruda Marques; **Unidade de Endocrinologia Pediátrica da Santa Casa de Misericórdia de São Paulo**: Luiz Eduardo Calliari*, Renata Maria de Noronha; **Instituto da Criança do Hospital das Clínicas da Universidade de São Paulo**: Thais Della Manna*, Roberta Salvodelli, Fernanda Garcia Penha; **Hospital das Clínicas da Faculdade de Medicina de Ribeirão Preto – USP**: Milton Cesar Foss*, Maria Cristina Foss-Freitas; **Ambulatório da Faculdade Estadual de Medicina de São José do Rio Preto**: Antonio Carlos Pires*, Fernando Cesar Robles; **Associação de Diabéticos de Bauru**: Carlos Antonio Negrato*, Maria de Fatima Guedes; **Centro de Diabetes da Escola Paulista de Medicina**: Sergio Atala Dib*, Patricia Dualib; **Clínica de Endocrinologia da Santa Casa de Belo Horizonte Setor Diabetes Tipo 1**: Saulo Cavalcanti da Silva*, Janice Sepulveda; **Ambulatório Multiprofissional de Atendimento à Diabetes do Hospital de Clínicas da Universidade Estadual de Londrina**: Henriqueta Guidio de Almeida*, Emerson Sampaio; Hospital de Clínicas da Universidade Federal do Paraná:Rosangela Roginski Rea*, Ana Cristina Ravazzani de Almeida Faria; **Instituto da Criança com Diabete Rio Grande Sul**: Balduino Tschiedel*, Suzana Lavigne, Gustavo Adolfo Cardozo; **Hospital de Clínicas de Porto Alegre**:Mirela Azevedo*, Luis Henrique Canani, Alessandra Teixeira Zucatti; **Hospital Universitário de Santa Catarina**: Marisa Helena Cesar Coral*, Daniela Aline Pereira; **Instituto de Diabetes-Endocrinologia de Joinville**: Luiz Antonio de Araujo*; **Hospital Regional de Taguatinga, Brasília**: Hermelinda Cordeiro Pedrosa*, Monica Tolentino; Flaviene Alves Prado; **Hospital Geral de Goiânia**: **Dr Alberto Rassi**: Nelson Rassi*, Leticia Bretones de Araujo; **Centro de Diabetes e Endocrinologia do Estado da Bahia**: Reine Marie Chaves Fonseca*; Alexis Dourado Guedes, Odelisa Silva de Mattos; **Universidade Federal do Maranhão**: Manuel Faria*, Rossana Azulay; **Centro Integrado de Diabetes e Hipertensão do Ceará**: Adriana Costa e Forti*, Cristina Façanha; **Universidade Federal do Ceará**: Renan Montenegro Junior*, Ana Paula Montenegro; **Universidade Federal de Sergipe**: Naira Horta Melo*, Karla Freire Rezende; **Hospital Universitário Alcides Carneiro**: Alberto Ramos *; **Hospital Universitário João de Barros Barreto, Pará**: João Soares Felicio *, Flavia Marques Santos; **Hospital Universitário Getúlio Vargas, Hospital Adriano Jorge**: Deborah Laredo Jezini*.

## Abbreviations

CV: cardiovascular; T1D: type 1 diabetes; sBP: systolic blood pressure; dBP: diastolic blood pressure; BMI: body mass index; HbA_1_c: glycated hemoglobin; T2D: type 2 diabetes; FPG: fasting plasma glucose; PPG: postprandial glucose; SBGM: self-blood glucose monitoring; HDL: high-density lipoprotein; LDL: low-density lipoprotein; NGSP: National Glycohemoglobin Standardization Program; CSII: continuous subcutaneous insulin infusion; ACE: angiotensin-converting-enzyme; BrazDiab1SG: Brazilian Type 1 Diabetes Study Group; DCCT: Diabetes Control and Complications Trial; EDIC: Epidemiology of Diabetes Interventions and Complications Trial.

## Competing interests

The authors declare that they have no competing interests.

## Authors’ contributions

MBG wrote, reviewed and edited the data. ASMM, RAC, LT and CAN reviewed the manuscript and researched the data. The investigators indicated by an asterisk and the program coordinators, the names of which are underlined, have collected and reviewed the research data. All authors read and approved the final manuscript.
